# VirPool: model-based estimation of SARS-CoV-2 variant proportions in wastewater samples

**DOI:** 10.1186/s12859-022-05100-3

**Published:** 2022-12-19

**Authors:** Askar Gafurov, Andrej Baláž, Fabian Amman, Kristína Boršová, Viktória Čabanová, Boris Klempa, Andreas Bergthaler, Tomáš Vinař, Broňa Brejová

**Affiliations:** 1 Faculty of Mathematics, Physics and Informatics, Comenius University in Bratislava, Bratislava, Slovakia; 2 CeMM Research Center for Molecular Medicine, Austrian Academy of Sciences, Lazarettgasse 14 AKH BT 25.3, 1090 Vienna, Austria; 3 Institute of Hygiene and Applied Immunology, Center for Pathophysiology, Infectiology and Immunology, Medical University of Vienna, Kinderspitalsgasse 15, Vienna, 1090 Austria; 4 Biomedical Research Center, Slovak Academy of Sciences, Bratislava, Slovakia

**Keywords:** SARS-CoV-2, Wastewater analysis, Variant proportion estimation, Probabilistic modeling, Weighted mixture model

## Abstract

**Background:**

The genomes of SARS-CoV-2 are classified into variants, some of which are monitored as variants of concern (e.g. the Delta variant B.1.617.2 or Omicron variant B.1.1.529). Proportions of these variants circulating in a human population are typically estimated by large-scale sequencing of individual patient samples. Sequencing a mixture of SARS-CoV-2 RNA molecules from wastewater provides a cost-effective alternative, but requires methods for estimating variant proportions in a mixed sample.

**Results:**

We propose a new method based on a probabilistic model of sequencing reads, capturing sequence diversity present within individual variants, as well as sequencing errors. The algorithm is implemented in an open source Python program called VirPool. We evaluate the accuracy of VirPool on several simulated and real sequencing data sets from both Illumina and nanopore sequencing platforms, including wastewater samples from Austria and France monitoring the onset of the Alpha variant.

**Conclusions:**

VirPool is a versatile tool for wastewater and other mixed-sample analysis that can handle both short- and long-read sequencing data. Our approach does not require pre-selection of characteristic mutations for variant profiles, it is able to use the entire length of reads instead of just the most informative positions, and can also capture haplotype dependencies within a single read.

**Supplementary Information:**

The online version contains supplementary material available at 10.1186/s12859-022-05100-3.

## Introduction

The pandemic of COVID-19 is accompanied by an unprecedented level of genomic surveillance of the severe acute respiratory syndrome coronavirus 2 (SARS-CoV-2), with more than 13.7 million genomic sequences deposited in the GISAID database [1] by early November 2022. These sequences are mostly obtained from single-patient samples following a positive clinical test. Long-term studies have shown that the composition of viral RNA fragments in wastewater reflects qualitatively and quantitatively the breakdown of virus lineages circulating in the population of the catchment [2, 3]. Wastewater-based epidemiology (WBE) has recently emerged as a cost effective and scalable alternative to sequencing individual sample patients [4, 5]. Various PCR-based techniques can be used to estimate virus gene copy numbers in wastewater to ascertain epidemiological trends. This can be accompanied by sequencing the mixture of virus genomes present in the sample, effectively mapping the sequence variation of the virus within a local population. Wastewater monitoring can help to address biases in analysis of clinical samples that are due to differences in test availability and willingness to undergo a clinical test. Moreover, increases in wastewater viral RNA levels can precede the results of clinical testing by several days or even longer when clinical testing is not readily available [5, 6], and thus wastewater analysis can provide early warning signals of worsening epidemic situation or emergence of new variants of the virus in a particular area. To this effect, wastewater based epidemiology has proven to be a complementary and independent perspective on the pandemic situation, valuable for public health authorities and their pandemic management efforts [7–9].

The World Health Organization monitors prevalence of virus variants around the world and selects variants of concern (VOCs) characterized by an increased transmissibility, virulence, or the ability to evade protection provided by vaccines and drugs. Several of these variants have caused massive epidemic waves, most notably the Alpha, Delta, and Omicron variants (Pango lineages B.1.1.7, B.1.617.2, and B.1.1.529, respectively). It is therefore of high interest to monitor the prevalence of these variants, particularly at an onset of a new wave, when the public health authorities expect the arrival of a new variant in a certain area.

Early work in analysis of SARS-CoV-2 wastewater samples concentrated on producing an overall consensus sequence of the sample or on detection of individual mutations followed by manual analysis of the results [2, 11–16]. Later, the presence of variants of concern was detected based on pre-selected mutations typical for each variant [17–19]. Pre-selected sites with mutations characteristic for individual variants were later also used to quantify the variant prevalence. At each such site, the proportion of the allele belonging to the variant is estimated, and the final proportion is determined as a mean or a median of single-site estimates [20–23]. Since each variant is considered independently of others, the method can produce inconsistent estimates (e.g. the sum of proportions of individual variants is greater than one) and can be biased by mutations shared by multiple variants, an issue which is likely to be exacerbated given the increased occurrence of convergent evolution events between different lineages due to selective pressures. To account for these shared mutations, Ellment et al. [24] estimated the proportions of variants by optimising the L2 metric between a mixture of base frequencies of individual variants and observed frequencies of specific mutation sites. Amman et al. [3] pushed this idea further by estimating the proportions jointly for multiple samples, taking the time of their collection into account. Such an approach could also be extended to account for geographical dependencies.

A similar problem was previously addressed in the context of virus populations. In the quasispecies spectrum reconstruction (QSR) problem, the aim is to analyze a sequencing sample containing reads from several distinct virus variants (also called haplotypes) to recover individual haplotype sequences and quantify their prevalence [25–27]. Typically, haplotypes are recovered by specialized assembly algorithms employing read overlaps and sequence coverage (see e.g. the path cover approach in ShoRAH [26]), followed by quantification of individual variants. In general, these approaches assume that individual haplotypes yield a consistent coverage across the whole reference genome, that the sequencing reads are randomly sampled from the haplotypes, and that haplotypes themselves are well represented by a single consensus sequence, possibly with a few local variations attributable to sequencing errors.

However, these assumptions are not satisfied in the case of SARS-CoV-2 wastewater sequencing. SARS-CoV-2 wastewater samples are typically sequenced by ARTIC protocol, originally developed in the context of Zika virus epidemics [28]. The virus sequence is divided into overlapping segments (called *amplicons*) that are first amplified through PCR, so as to increase the number of molecules present in the sequencing sample. Depending on the protocol, the approximate length of amplicons varies between 400bp [29] (further referred to as *short amplicons*) and 1.5-2.5kbp [30–32] (*long amplicons*). Only after the PCR amplification, the pooled sample is sequenced by Illumina or Oxford Nanopore MinION sequencers. The efficiency of the PCR amplification varies between amplicons, resulting in highly uneven coverage along the genome. Moreover, sequencing reads do not span amplicon boundaries, breaking the haplotype linkage between the amplicons. Finally, individual SARS-CoV-2 variants typically include a large number of distinct sequences, and this diversity is difficult to express as a single consensus sequence.

In this paper, we introduce a new approach based on a probabilistic model of sequencing reads originating from a mixture of variants. Our model captures sequence diversity present within individual variants through employing *variant profiles* derived from available GISAID sequences for a particular variant. At the same time, we also model sequencing errors, which is essential in application to data sets obtained by sequencing technologies with higher error rates, such as nanopore sequencing. Our approach does not classify individual reads or sites as belonging to a particular variant, but instead searches for a solution that has the highest consistency with the observed data. Consequently, we do not require pre-selection of sites characteristic for each variant, and we can use the information contained in the full length of the sequenced reads. In this aspect, our approach is similar to the approach by Eriksson et al. [25], though our model is more complex due to the specifics of the wastewater analysis problem. Finally, our approach is able to exploit linkage between individual sites within the same sequencing read, which leads to an increased accuracy in case of using long nanopore amplicons, in spite of higher sequencing error rates of nanopore sequencing. We tested our software on analysis of both simulated and real data sets, and we showed that our approach outperforms the median approach previously employed for estimating proportions of SARS-CoV-2 variants in wastewater samples.

## Results

### Mixture model for variant proportion estimation

For a given sequencing sample, VirPool estimates the fraction of sequencing reads originating from selected variants. Variant *k* is characterized by its *variant profile* $P_k$, where $P_{k}(i, a)$ is the probability of observing nucleotide *a* at position *i* in variant *k*. The positions are numbered according to the reference genome, and all sequences that we consider are aligned to this reference genome.

The VirPool algorithm is governed by a probabilistic model that assigns a likelihood to weights of individual variants $w_1,\dots ,w_K$, where *K* is the number of variant profiles. Intuitively, these weights correspond to proportions of sequencing reads originating from individual variants mixed in a particular sample. Assuming that a particular read $R=r_1r_2\dots r_{|R|}$ starting at position *s* originates from a variant *k*, the probability of observing this read is simply

1 $$\begin{aligned} \Pr (R|k) = \prod _{i=1}^{|R|} P_k(s+i-1,r_i). \end{aligned}$$

This probability needs to be also adjusted for sequencing errors (for details, see Methods).

The overall likelihood is the probability of observing sequence of reads $\rho =(R_1,\dots ,R_m)$ given the variant weights $w_1,\dots ,w_K$. In our model, each read is generated independently; thus the likelihood can be expressed as

2 $$\begin{aligned} L(w_1,\dots ,w_K\,|\,\rho ) = \Pr (\rho \,|\,w_1,\dots ,w_K) = \prod _{i=1}^m \sum _{k=1}^K w_k \Pr (R_i|k). \end{aligned}$$

Here, we assume that starting positions and lengths of individual reads are fixed in advance. Note that if these were sampled from any distribution independent of variants, this would only add a constant factor to the likelihood and would have no influence on the weight optimization.

For a given set of sequencing reads and variant profiles, the weights are estimated so that the likelihood $L(w_1,\dots ,w_K|\rho )$ is maximized. For details of the optimization algorithm, see Methods.

The results of VirPool analysis are dependent on the selection of variants included in the analysis. In our work, we rely on Pango lineage classification of sequenced virus genomes [10], which is based on phylogenetic analysis. Selected clades of the phylogenetic tree are assigned Pango lineage identifiers, leading to a hierarchical system of subclades nested in larger clades. We use a selection of several Pango lineages as virus variants in our analysis. Typically, one would select variants of interest circulating in a given region at a considered time. In our experiments, we also include variant “other” that represents all the remaining SARS-CoV-2 sequences not belonging to the selected variants. A high weight of the “other” variant in the result allows the user to detect that the set of the variants should be adjusted.

### Accurate prediction of variant proportions for different sequencing technologies

To evaluate the accuracy of our methods, we prepared synthetic mixtures combining several single-patient sequencing read sets downloaded from public databases, each containing sequencing reads from a single virus variant. We selected four variants common in Europe in the fall of 2020 (B.1.1.7, B.1.160, B.1.177, B.1.258), as well as samples from other variants which should be correctly classified as “other” profile (B.1.221, B.1.1.170, B.1.367, B.1.1.37, AP.1); see Methods for the mixture creation details. We estimated proportions of these profiles both by VirPool and by the baseline median method described in Methods and compared the results to true proportions.

**Table 1:** Comparison of true and estimated variant proportions for the synthetic mixtures

Mixture name		B.1.1.7	B.1.160	B.1.177	B.1.258	Other
Ont-long-1	True	0.19	0.42	0.23	0.16	0.00
	VirPool	0.18	0.42	0.23	0.17	0.00
	Median	0.19	0.43	0.24	0.17	− 0.03
Ont-long-2	True	0.17	0.38	0.21	0.15	0.09
	VirPool	0.16	0.38	0.22	0.15	0.09
	Median	0.17	0.38	0.20	0.15	0.09
Ont-long-3	True	0.42	0.00	0.00	0.35	0.23
	VirPool	0.41	0.00	0.00	0.37	0.22
	Median	0.39	0.01	0.02	0.36	0.23
Ont-long-4	True	0.00	0.65	0.35	0.00	0.00
	VirPool	0.00	0.64	0.36	0.00	0.00
	Median	0.01	0.63	0.43	0.02	− 0.08
Ont-short-1	True	0.19	0.42	0.23	0.16	0.00
	VirPool	0.18	0.42	0.23	0.17	0.00
	Median	0.19	0.43	0.24	0.17	− 0.03
Ont-short-2	True	0.17	0.38	0.21	0.15	0.09
	VirPool	0.16	0.38	0.22	0.15	0.09
	Median	0.17	0.38	0.20	0.15	0.09
Ont-short-3	True	0.42	0.00	0.00	0.35	0.23
	VirPool	0.41	0.00	0.00	0.37	0.22
	Median	0.39	0.01	0.02	0.36	0.23
Ont-short-4	True	0.00	0.65	0.35	0.00	0.00
	VirPool	0.00	0.64	0.36	0.00	0.00
	Median	0.01	0.63	0.43	0.02	-0.08
Illumina-1	True	0.15	0.23	0.27	0.34	0.00
	VirPool	0.16	0.26	0.29	0.29	0.00
	Median	0.20	0.26	0.30	0.35	− 0.11
Illumina-2	True	0.13	0.20	0.23	0.29	0.16
	VirPool	0.14	0.22	0.25	0.25	0.13
	Median	0.17	0.23	0.25	0.30	0.06
Illumina-3	True	0.16	0.25	0.00	0.37	0.21
	VirPool	0.18	0.29	0.01	0.33	0.19
	Median	0.23	0.32	0.00	0.38	0.07
Illumina-4	True	0.00	0.46	0.54	0.00	0.00
	VirPool	0.00	0.47	0.53	0.00	0.00
	Median	0.00	0.49	0.66	0.00	− 0.16

The first group corresponds to mixtures created with Oxford Nanopore reads with long (2 kbp) amplicons, the second group corresponds to mixtures created with Oxford Nanopore reads with short (400 bp) amplicons, and the third group corresponds to mixtures created with Illumina reads. The proportion of other for the median estimator is calculated as 1 minus the sum of the estimated proportions (therefore it can be negative)

Table 1 shows that in almost all cases, VirPool can accurately estimate the true proportions. This is true for all three considered sequencing protocols (Illumina paired 150bp reads with 400bp amplicons, Oxford Nanopore short 400bp amplicons, and Oxford Nanopore long 2kbp amplicons). The method also worked well when the mixtures contained variants included in the “other” combined profile. The median method [21], while providing in many cases similar results to VirPool, systematically overestimates the explicitly listed variants, in many cases resulting in estimates of named variants summing to more than 1.

**Fig. 1:**
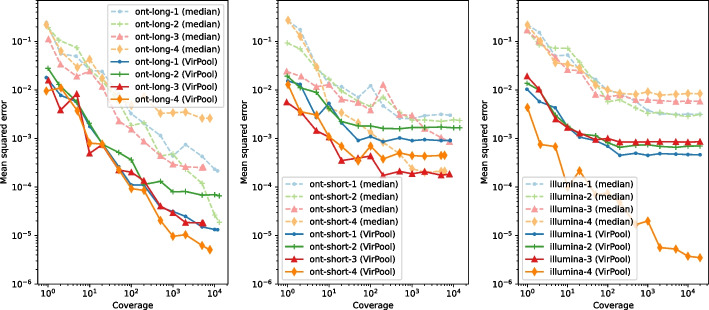
Variant proportion estimation errors as a function of sequencing coverage. Left: long nanopore mixtures; Center: short nanopore mixtures; Right: Illumina mixtures. The mean squared error (MSE) was averaged over ten random subsamples of synthetic mixtures from Table 1 for each considered coverage. Both axes are in logarithmic scale

### Prediction accuracy at low coverages

To evaluate the accuracy at lower coverages, we subsampled our synthetic mixtures. Figure 1 shows the mean squared error of predictions averaged over multiple subsamples. Even though nanopore sequencing has a much higher sequencing error rate than Illumina, the best weight estimates are achieved with nanopore long 2kb amplicons. This suggests that VirPool can effectively take advantage of long-range dependencies between positions covered by a single amplicon. For both nanopore and Illumina with short amplicons, the error decreases with increasing coverage until reaching a plateau, in most cases around $100\times$ genome coverage. In most cases, the median approach has significantly larger error than VirPool.

**Fig. 2:**
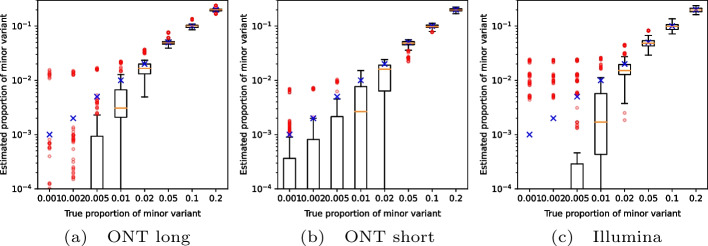
Estimated proportion of the minor variant in synthetic experiments among fall 2020 samples. Blue crosses represent the true proportion of the minor variant. Orange lines represent the median, red circles are outliers. In each setting, the graphs show the distribution for 10 randomly generated data sets for each pair of variants (including the “other” group). The average coverage for each synthetic data set was 5000

### Reliability of detection of low-abundance variants

To test detection of variants occurring at low frequencies, we created artificial mixtures of two variants from among the fall of 2020 samples, with the minor variant present in frequencies ranging from 0.1 to 20%. Fig. 2 shows that VirPool is generally accurate for frequencies of 5% or more. Even frequencies of 2% were generally detected, but the variance in the results is high. Conversely, at very low frequencies, the presence of the minor variant may be overestimated. Note that detection of such low-frequency variants has been shown to be difficult and requires an extremely high coverage even with Illumina sequencing data [34].

**Fig. 3:**
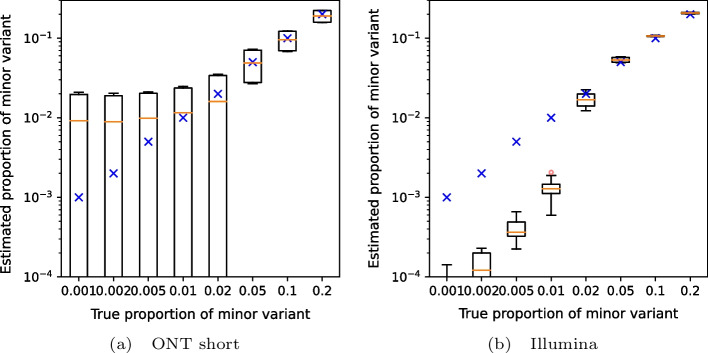
Estimated proportion of the minor variant in synthetic mixtures of the Alpha and Delta variants. Blue crosses represent the true proportion of the minor variant. Orange lines represent the median, red circles are outliers. In each setting, the graphs show the distribution for 20 randomly generated data sets with the prescribed minor variant proportion, with Alpha and Delta playing the role of the minor variant in 10 samples each. The average coverage for each synthetic data set was 5000

**Fig. 4:**
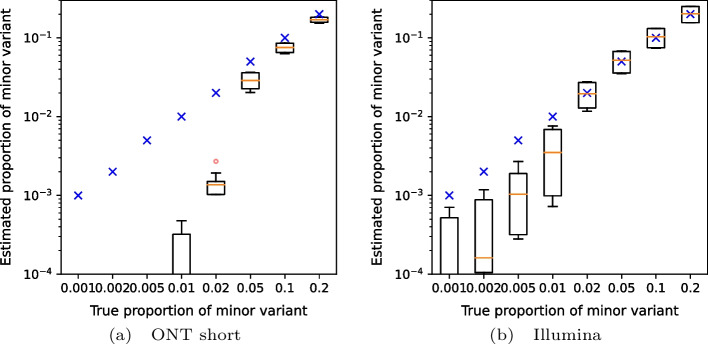
Estimated proportion of the minor variant in synthetic mixtures of the Delta and Omicron variants. Blue crosses represent the true proportion of the minor variant. Orange lines represent the median, red circles are outliers. In each setting, the graphs show the distribution for 20 randomly generated data sets with the prescribed minor variant proportion, with Delta and Omicron playing the role of the minor variant in 10 samples each. The average coverage for each synthetic data set was 5000

We also attempted to replicate data corresponding to the Alpha (B.1.1.7) wave transitioning to Delta (B.1.617.2) (Fig. 3) and Delta transitioning to Omicron (B.1.1.529, specifically BA.1 subvariant) (Fig. 4). Again, results above 5% are reliable, with the exception of short nanopore data in the Omicron wave, where the estimated weights of Omicron are lower due to a false prediction of the “other” variant.

**Table 2:** Estimated proportions for the in-vitro mixture sample of eight patient samples

	B.1.1.7	B.1.160	B.1.177	B.1.258	other
Number of samples	2 samples	1 sample	0 samples	4 samples	1 sample
Estimate from unique mutations	0.55	0.08	0.00	0.33	0.07
VirPool estimate	0.53	0.07	0.00	0.25	0.15

The second rows shows a simple estimate based on computing the median of frequencies for mutations unique to one of the eight samples and then summing the medians over the samples with the same variant

### In-vitro mixture of patient samples

Using nanopore long amplicons, we sequenced and analyzed a mixture of eight clinical samples. Presence or absence of individual variants was correctly identified by VirPool (Table 2), and the estimated proportions agree well with a separate analysis based on examination of frequencies of mutations specific for individual clinical samples, as determined by previous sequencing of each sample individually. Even though an effort has been made to balance the original sample proportions using dilution factors based on measured Cq values (Additional file 1: Table S2), the resulting proportions are influenced by many factors, such as different levels of fragmentation of RNA and subsequent differences in amplification efficiency.

### Analysis of wastewater samples from the Alpha wave

We applied VirPool to time series data sets of wastewater samples spanning several months in regions of Bischofshofen, Austria (Illumina data) [3] and Nice, France (nanopore data) [21]. Wastewater samples are highly challenging, because the coverage is often highly uneven (see Additional file 1: Fig. S1).

**Fig. 5:**
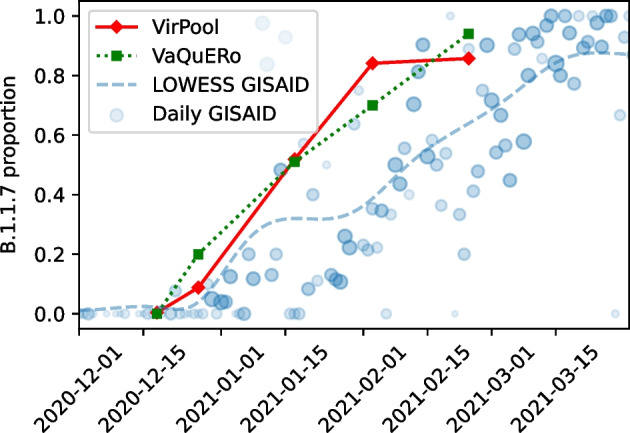
Estimated proportions of the Alpha (B.1.1.7) variant in wastewater samples from Bischofshofen, Austria [3]. The red line represents the proportions estimated by VirPool model. The green line represents the proportions estimated by VaQuERo model [3]. The blue circles represent the daily proportions of B.1.1.7 among Austrian samples submitted to GISAID, the number of samples is reflected by circle size; blue line is the smoothed version of those data

Figure 5 shows the analysis of a time series sampled between December 2020 and February 2021 from the area of Bischofshofen, Austria (State of Salzburg), sequenced by Illumina short read protocol. Comparing VirPool analysis to the analysis by VaQuERo pipeline [3], both tools predict a sharp increase in the Alpha variant in January and February of 2021, which is also apparent in the clinical samples from GISAID.

Both methods also agree on the general composition of samples and most of the trends (Additional file 1: Table S4). One of the notable differences is a lower prevalence of the Alpha variant at the end of February in VirPool predictions, which is compensated by a rebound in the B.1.258 prevalence to 10%. By examining allele frequencies, we found 7 alleles that strongly support inclusion of B.1.258 (Additional file 1: Table S5); three of these alleles are typical for the whole B.1.258 clade, while four additional alleles are characteristic for subvariant B.1.258.17, which was indeed detected in clinical samples in the state of Salzburg (12 samples out of 136 in GISAID in February and March). In contrast, several alleles very common in B.1.258 are either missing completely (e.g., 8047T) or are present at very low frequencies (e.g., 7767C, 22879A, 29734C). This may be a consequence of a very uneven coverage or variant-specific differences in amplification efficiency for specific primers. It may possibly also indicate recombination or some other variant sharing characteristic mutations with B.1.258.17; ten samples in GISAID outside of B.1.258 collected between January and March share the same 7 alleles. Their classification is typically generic (B.1), while some of them are classified as B.1.367 or B.1.221. In spite of this uncertainty, the data seem to support a lower prevalence of the Alpha variant and a possible presence of B.1.258.17 variant in agreement with the VirPool predictions.

**Fig. 6:**
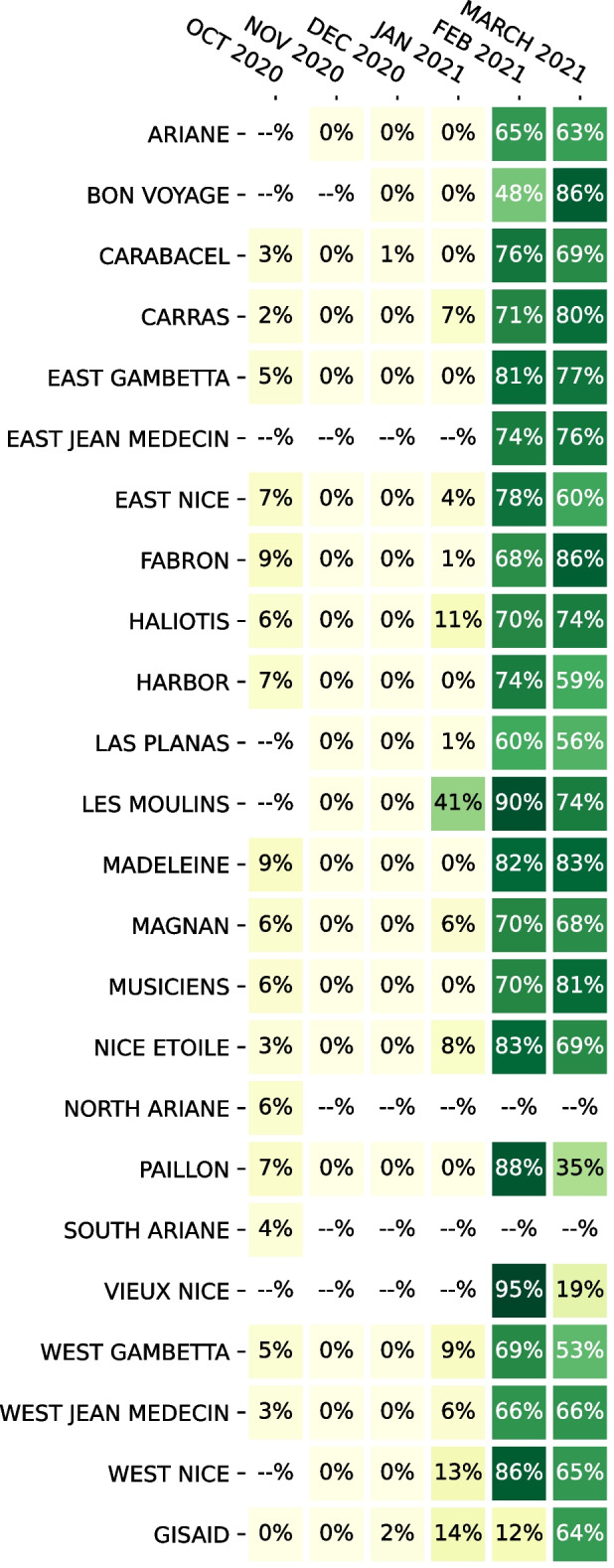
Estimated the Alpha (B.1.1.7) proportions in wastewater samples from Nice, France, using data from Rios et al. [21]. If multiple samples were sequenced for a given location and month, the median was taken. The last row (“GISAID”) is the proportion of sequences with variant B.1.1.7 submitted to GISAID database from French region Provence-Alpes-Côte d’Azur in the same month

One of the strengths of VirPool is that the same method can be applied to both Illumina and nanopore data sets, only changing settings for the sequencing error rate. Figure 6 shows the result of analysis of selected samples from Nice, France, sequenced by the short-amplicon nanopore sequencing protocol. In agreement with Rios et al. [21], we observe a very sharp increase in the Alpha variant prevalence in February 2021, which is not observed in the clinical samples from GISAID in the Provence-Alpes-Côte d’Azur region, suggesting that this outbreak was not captured in a timely manner by genome sequencing efforts. In agreement with Rios et al. [21], we see a significant prevalence of the Alpha variant in the Les Moulins site already in January 2021.

VirPool consistently predicts several percent of the Alpha variant in all samples from October 2020. Rios et al. [21] also observe several mutations characteristic for the Alpha variant in October 2020, but do not comment or investigate this phenomenon. Upon closer examination, we see a similar pattern as in the case of B.1.258 variant in the Austrian samples, with some mutations characteristic for a given variant (in this case Alpha) having a relatively high frequency, while others being absent (Additional file 1: Table S6). Here, having samples from multiple locations, we observe that some genome positions give consistent results, while others vary between samples, further supporting the hypothesis of amplification efficiency differences. Overall, we hypothesize that the Alpha variant was indeed present in the area, which is further supported by three GISAID Alpha samples collected in Marseille in the same month.

VirPool also estimates a significant decrease in the Alpha variant proportions in several locations between February and March 2021. We investigated this decrease in the Harbor location (from 74% in February to 59% in March), where VirPool estimates a rebound of variants B.1.177 (to 11%) and B.1.160 (to 15%). The predicted increase in B.1.177 seems to be driven by C22227T mutation, which is characteristic for B.1.177 and very rare in B.1.1.7; nonetheless, it appeared at the frequency of 69% in the reads from this sample (Additional file 1: Table S7). All the other mutations characteristic for B.1.177 are either present at much lower frequencies or completely missing. Also, C22227T mutation occurs in several B.1.1.7 GISAID samples from France. All this evidence leads us to a conclusion that a B.1.1.7 subvariant with this mutation has been highly prevalent in this area and the rebound of B.1.177 is a false positive. This points to a weakness of variant characterization by probabilistic profiles representing global distribution of mutations, which may not agree with locally circulating strains, although as we see in other experiments, this usually does not cause problems in the estimation.

## Methods

### Variant profile estimation

Our method requires a set of *K* profiles, each representing one variant of the SARS-CoV-2 virus. In these profiles, $P_{k}(i, a)$ is the probability of observing nucleotide *a* at position *i* in variant *k*, where positions are numbered according to the reference sequence (we use Wuhan/Hu-1/2019). In our experiments, we build these profiles from the SARS-CoV-2 genomic sequences downloaded from the GISAID database [1]. We used GISAID version from February 5, 2022, omitting sequences with incomplete or missing collection date, and incomplete genomes with less than 25kbp of sequence. We then subsampled the data so that at most roughly 50,000 sequences were kept per month. In specific experiments, we use only a subset of GISAID records corresponding to the period from which our samples originate.

In each experiment, we selected several variants designated by their Pango lineage identifier (Additional file 2, part B). The profile for each selected variant is built from the samples assigned to this lineage and its sublineages according to GISAID metadata. Specifically, we assign each sequence to the nearest ancestor clade from our list of selected lineages. This allows for selecting both a lineage and its sublineage, such as B.1.1 and B.1.1.7; in such case sequences from the sublineage are excluded from the parent lineage profile. If no ancestor clade is in the list, the sequence is assigned label “other”, representing genomic background of all other lineages.

Each sequence was aligned to the common reference sequence Wuhan/Hu-1/2019 by minimap2 [35]. Intuitively, value $P_{k}(i, a)$ would be estimated from data as the relative frequency of symbol *a* at position *i* among the genomes assigned to the variant *k*. However, some variants contain characteristic deletions shared by almost all genomes belonging to the variant. As a result, some genomic positions are covered by only a small number of genomes belonging to variant *k*. Let $\gamma _k$ be a threshold on the coverage. Then we set the variant profile probabilities as follows:

$$\begin{aligned} P_k(i, a) = \frac{C_k(i, a)}{\max \left\{ \gamma _k, \sum _{b} C_k(i, b)\right\} }, \end{aligned}$$

where $C_k(i, a)$ is the number of occurrences of base *a* at position *i* among genomes of variant *k*. Thus, the positions with coverage lower than $\gamma _k$, typically containing gaps characteristic for the variant, will have the sum of values $P_k(i,a)$ lower than one. This will have no impact on reads originating from variant *k*, because they have gaps at such positions, but it will penalize reads from other variants that do not have such gaps. In our experiments, we set $\gamma _k$ as the coverage at the first percentile (smallest 1%) of coverage within the genome for each variant *k*.

To select the list of variants for analysis of new samples, the user would choose ones that circulate in a particular area at the time, with additional variants added from relevant watch lists (such as WHO variants of concern and variants of interest). The profiles should be built based on sequences with recent collection dates (latest months) and updated periodically in order to include recent evolutionary changes within virus sequences.

### Substitution error

The mixture model characterized by equations (1) and (2) in the Results section assumed error-free sequencing. In our final model, we add substitution sequencing errors that occur uniformly at random with error rate $\varepsilon$. The probability of observing read $R=r_1\dots r_{|R|}$ at position *s* from variant *k* is then

$$\begin{aligned} \Pr [R|k, \varepsilon ] = \prod _{i = 1}^{|R|}~\left[ \left( 1 - \varepsilon \right) \cdot P_k(s + i - 1, r_i) + \frac{\varepsilon }{3} \cdot \sum _{a \ne r_i} P_k(s+i-1,a)\right] \end{aligned}$$

In our experiments, we use $\varepsilon =0.001$ for Illumina reads and $\varepsilon =0.05$ for nanopore reads. Note that both real insertions and insertion errors are ignored by our model; read positions with a deletion are treated as missing data.

### Mixture weight estimation

The optimal weights $W=w_1,\dots , w_K$ for a read set $\{R_1,\dots , R_m\}$ are estimated via minimisation of the negative log-likelihood

$$\begin{aligned} W^* = \underset{\begin{array}{c} w_k \ge 0,\\ \sum w_k = 1 \end{array}}{\arg \min }~-\sum _{i=1}^m~\log \left[ \sum _{k=1}^K w_k \cdot \Pr [R_i|k]\right] \end{aligned}$$

To simplify the optimisation task to an unconstrained case, we use *softmax transformation* [36]. Namely, we define auxiliary variables $\xi _1, \ldots , \xi _K$ and set the values of the original weight variables $w_1, \ldots , w_K$ as the softmax of the auxiliary variables:

$$\begin{aligned} w_i = \frac{e^{\xi _i}}{\sum _{j=1}^K e^{\xi _j}}, \end{aligned}$$

This transformation ensures that all weight variables are non-negative and sum up to 1. This leads to the following unconstrained optimization task:

$$\begin{aligned} W^* = \text {softmax}\left\{ \underset{\xi _1, \ldots , \xi _K}{\arg \min }~-\sum _{i=1}^m~\log \left[ \sum _{k=1}^K \frac{e^{\xi _k}}{\sum _{j=1}^K e^{\xi _j}} \cdot \Pr [R_i|k]\right] \right\} . \end{aligned}$$

The minimisation is done using the L-BFGS-B algorithm [37] implemented in scipy Python library [38] with symbolic Jacobian. We use numba library to speed up the calculations by pre-compilation of time-critical functions.

### Analysis of sequencing samples

Alignments of sequencing reads to the reference genome were downloaded from ENA, or where needed, were produced by minimap2 for ONT reads and bwa-mem for Illumina reads. We use only primary alignments for each read. In our analysis, we consider a paired read as a single unit with both parts originating in the same variant, and we require that both parts are aligned.

Some mutations tend to occur repeatedly within the virus evolution (homoplasic sites). To avoid confusion between variants, we mask known homoplasic sites [39] and do not consider them in the analysis. Some variants have mutations within primer binding positions. These positions will appear in the reads as matching the primer, not the sequenced sample. Since in most cases, these primers are not trimmed in the underlying data sets, we mask primer positions (ARTIC protocol V3 or V4 as appropriate).

### Baseline method and evaluation

In our experiments, we compare VirPool with an estimator based on median proportions previously used by Rios et al. [21]; similar methods were also used by other studies [20, 22, 23]. For each selected variant, this estimator needs a list of characteristic mutations; we use the lists by Rios et al. [21] from their code repository [40]. The relative frequency of each characteristic mutation is computed among reads aligned to the corresponding position. The resulting estimator of the variant’s proportion is the median of these frequencies. Since the median estimate is computed independently for each variant, it is not guaranteed that proportions of all variants will sum to one. We attribute the remaining probability to the background “other” variant; this can also be a negative number.

To evaluate the accuracy of both VirPool and the median estimator on simulated data, we use the *mean squared error* (*MSE*) measure defined as $\textit{MSE}(W, {\hat{W}}) = \frac{1}{K} \cdot \sum _{k=1}^K \left( w_k - {\hat{w}}_k\right) ^{2}$, where $W = (w_1, \ldots , w_K)$ and ${\hat{W}} = ({\hat{w}}_1, \ldots , {\hat{w}}_K)$ are the true and estimated proportions of variants, respectively. Note that this metric supports negative weight estimates, so it can be used for the median estimator.

### Synthetic mixtures

The synthetic mixtures for evaluating the accuracy of our method were created by combining reads from several single-patient sequencing samples downloaded from ENA (see Additional file 2 parts A and B). For initial experiments, we selected a subset of considered variants and pooled all mapped reads from all fall 2020 samples belonging to these variants (Additional file 2, part A). For experiments evaluating the accuracy at low coverages, we subsampled these mixtures to the desired coverage. To assess the sensitivity to low abundance variants, we created mixtures of pairs of variants, again pooling all samples belonging to a particular variant from the fall 2020 samples. The reads corresponding to the two variants were then subsampled to achieve the desired proportions and the total average coverage of 5000. A similar procedure was used for samples representing transitions from Alpha to Delta and from Delta to Omicron. In all syntetic mixtures, the true proportions were defined as the proportion of reads originating from the samples belonging to a particular variant.

### In vitro mixture sequencing

We created and sequenced a mixture of eight single-patient samples (Additional file 1: Table S2) that were previously sequenced individually at the Biomedical Research Centre of the Slovak Academy of Sciences in Bratislava, Slovakia. The samples originated from oropharyngeal swabs collected in January 2021. The sample processing and sequencing library construction were carried out as described earlier [41], generally following the COVID-19 virus protocol (PTC_9096_v109_revF_06Feb2020; Oxford Nanopore Technologies, Oxford, UK) with some modifications. After RNA extraction for each sample, SARS-CoV-2 RNA was quantified by an RT-qPCR assay carried on QuantStudio™ 5 Real-Time PCR System (Applied Biosystem, Foster City, California, USA). Individual samples were diluted according to the obtained quantification cycle (Cq) to obtain approximately equimolar mixture (Additional file 1: Table S2). From this mixture of all eight samples, 11$\mu$l was then used for reverse transcription. The resulting cDNA was amplified using the 2-kb primer scheme [30], and the sequencing library was constructed using a ligation kit (SQK-LSK109). A single library was used for sequencing, and thus no barcoding was performed. The library was sequenced using an R9.4.1 flow cell (FLO-MIN106) on a MinION Mk-1b device (Oxford Nanopore Technologies, Oxford, UK). Nanopore sequencing data were base called using Guppy v.4.4.1 and aligned using minimap2 v.2.13-r852 [35].

### Wastewater samples

As some wastewater samples have a very uneven coverage, signal from extremely highly covered positions can overwhelm the rest of the genome. Therefore we subsample reads from such highly covered positions. In our experiments, we set coverage threshold *t* to 1000. For each read, we compute the median coverage $m_r$ of the genome in the area covered by this read. The read is then chosen into the subsampled set with probability $\min \{1, \frac{t}{m_r}\}$. As a result, all reads are kept in regions with coverage below *t*.

To visually compare the time series obtained by our wastewater analysis with variant proportions from GISAID (as in Fig. 5), we smooth the GISAID frequencies using *locally weighted linear regression* [42], as the number of samples sequenced in a single day in the location of interest can be low. The data set used for regression contains a point for each sample from a given location in GISAID. This point is (*d*, 1) for a sample from date *d* with the given variant or (*d*, 0) for any other variant. In other words, vector *X* consists of dates of the samples (converted to e.g. day difference from the beginning of year 2020), and vector *Y* contains values 0 and 1, depending on the variant of each sample. We use the Gaussian distance function $w(x, z) = \exp \left( -\frac{(x-z)^2}{2\tau ^2}\right) ,$ where $\tau$ is a smoothness parameter.

## Discussion

In this paper, we presented a new method for estimating the proportions of SARS-CoV-2 variants in mixed samples based on a probabilistic model, which captures known genetic variability of each variant. Explicit modeling of sequencing errors and utilization of haplotype information makes the proposed approach particularly useful for current long-read sequencing technologies. We demonstrated on synthetic mixtures that our tool gives accurate results for different sequencing technologies and with different variant combinations, including variants that were included in the background “other” profile. The proportions can be estimated even at relatively low coverage and even for variants with proportions as low as 5%. This makes it also relevant for deconvoluting coinfections in single-patient samples. Our tool can be easily adapted to other viruses where a comprehensive database of sequences belonging to individual clades is available.

Our work suggests several open problems in this area. First, we selected the set of variants manually in this paper. This is often practical, as various authorities post lists of variants of concern. Nonetheless, automated selection of relevant variants for a given sample is an interesting problem. Some work in this direction was done by Amman et al. [3] who select variants prior to estimating their proportions.

It would be also desirable to provide some measure of confidence whether a given variant actually appears in the sample, particularly when the model predicts relatively low proportion. This can be achieved by measuring of the statistical significance of individual estimated proportions, similarly to classic regression analysis, or by requiring that reads supporting a given variant are spread along the entire genome. Highly uneven support of a variant along the genome can be caused by the presence of recombinant viruses. It would be interesting to extend our model to discover the presence of such recombinants in the sample.

We could also extend our probabilistic model by removing various assumptions built into it. Although the entire read is in our model generated from one variant, and thus the positions in a read are not independent, once the variant is fixed, the bases in the variant profile are assumed to be independent. By building more complex variant profiles it would be possible to capture linkage between different genome positions, particularly positions within a single amplicon. Similarly, although we do not assume that the coverage is uniform at all positions, we assume that the variant proportions are the same at all positions in the genome. However, mutations occurring at primer binding sites may render some primer pairs less efficient in some variants, which violates this assumption (see e.g. changes in the ARTIC primer sets V4 and V4.1 to avoid mutations in the Delta and Omicron variants, respectively). To handle this phenomenon, our model can be extended to consider the starting position of the read as a random variable. The typical read depth profiles of individual variants necessary for this change can be estimated from single-patient samples sequenced using the same technology and primer set.

A crucial property of our model is its ability to capture long-range dependencies within reads, which is particularly relevant when coupled with use of long amplicons and nanopore sequencing. In fact, an early version of our model [33], which was unable to take this information into account, suffered from lower accuracy which had to be addressed by ad-hoc heuristics. The utilization of long-read sequencing technology can also by beneficial for the *de novo* characterisation of variants from wastewater sequencing data alone, which is still an open and challenging task. Furthermore, the usefulness of WBE critically depends on a timely availability of its results to health authorities and policymakers [7, 8]. The portability of Oxford Nanopore MinION devices potentially makes an on-site application feasible, drastically reducing logistical challenges. VirPool’s evidenced capability to use nanopore data thus may serve as a catalyst for a technology shift.

## Supplementary Information

**Additional file 1.** Supplementary tables and figures.

**Additional file 2.** (A) Overview of single-patient clinical samples used in synthetic mixtures. (B) Composition of synthetic mixtures and parameters of VirPool analysis. (C) Overview of wastewater samples.

## Data Availability

In vitro mixture sequencing data are available from European Nucleotide Archive under project number PRJEB53383. Data from other studies are available from European Nucleotide Archive through accession numbers listed in Additional file 1: Table S1. We gratefully acknowledge the authors from the originating laboratories responsible for obtaining the specimens, as well as the submitting laboratories where the genome data were generated and shared via GISAID (https://www.gisaid.org/) which were used to estimate variant profiles. VirPool is an open source software available at https://github.com/fmfi-compbio/virpool.
