# Supplementary material for: VirPool: model-based estimation of SARS-CoV-2 variant proportions in wastewater samples
Source: BMC Bioinformatics. 2022 Dec 19;23:551. doi: 10.1186/s12859-022-05100-3 (PMC9761630; doi:10.1186/s12859-022-05100-3)
Supplement: Supplementary file 1 — Additional file 1. Supplementary tables and figures. [file 12859_2022_5100_MOESM1_ESM.pdf]

VirPool: Model-Based Estimation of SARS-CoV-2 Variant Proportions in Wastewater Samples

Supplementary Tables and Figures

Table S1: (a) Overview of single-patient clinical samples used in synthetic mixtures. (b) Composition of synthetic mixtures and parameters of VirPool analysis. (c) Overview of wastewater samples.

This table is in the attached supplementary file 1.

Table S2: Eight single-patient samples mixed in the in vitro mixture. The dilution of each sample was computed based on the Cq value from RT-qPCR. Among mutations discovered in individual samples we have selected those that are specific to a single of these eight samples, and we report their count and the median frequency of these mutations in the mixed sample. The two B.1.1.7 samples have only a single unique mutation each, making their estimates less reliable, but their sum agrees well with proportions of mutations characteristic for this variant shown in Table S3).

Sample ID	GISAID	Variant	Cq	Dilution	Unique mut.	Freq.
UKBA-1122/2021	EPI_ISL_959634	B.1.1.7	15.924	14.1	1	0.243
UKBA-1123/2021	EPI_ISL_959635	B.1.1.7	15.915	14.2	1	0.307
UKBA-1102/2021	EPI_ISL_959642	B.1.160	16.873	7.3	24	0.084
UKBA-1118/2021	EPI_ISL_959631	B.1.258	16.292	10.9	8	0.009
UKBA-1108/2021	EPI_ISL_959649	B.1.258	19.738	1.0	11	0.023
UKBA-1124/2021	EPI_ISL_959636	B.1.258	16.748	7.9	10	0.129
UKBA-1103/2021	EPI_ISL_959648	B.1.258	16.348	10.5	7	0.165
UKBA-1105/2021	EPI_ISL_959647	B.1.1.170	17.681	4.2	19	0.065

Table S3: Frequencies of selected alleles in the reads from the in vitro mixed sample. We have selected all alleles which had in our variant profile probability more than 90% in B.1.1.7 and less than 1% in the other variants considered in the analysis, including the background "other" profile. We display the number and fraction of reads containing this allele and the probability in the B.1.1.7 profile. The median allele proportion is 0.524, which agrees well with the Virpool estimate of 53% for B.1.1.7. Most positions have a similar proportion of the B.1.1.7 allele, except for the last one.

			Frequency in	
Pos	Allele	# reads	reads	B.1.1.7
23271	A	12762	0.615	1.000
23709	T	12240	0.606	1.000
23063	T	12108	0.597	1.000
14676	T	39639	0.581	1.000
15279	T	38363	0.574	1.000
24506	G	11952	0.566	1.000
24914	C	11645	0.560	0.999
16176	C	11035	0.549	0.999
5388	A	24426	0.538	1.000
6954	C	10154	0.527	0.999
28111	G	31574	0.521	1.000
913	T	14904	0.502	1.000
3267	T	12638	0.489	1.000
28280	C	34572	0.487	0.998
27972	T	27369	0.486	1.000
28977	T	26171	0.486	1.000
28048	T	28749	0.484	0.998
28282	A	33981	0.477	0.998
28281	T	33260	0.445	0.998
11296	G	456	0.053	0.918

Table S4: The estimated proportions in wastewater samples from a plant near Bischofshofen, Austria (federal state of Salzburg) (Amman et al, 2022). Comparison of predictions with VirPool and VaQuERo (Amman et al, 2022). Column "other" for VaQuERo includes lineages B.1.221, B.1.1.232, and B.1.1.153 as well as other unidentified lineages.

Date	B.1.1.7	B.1.160	B.1.177	B.1.258	B.1.351	other
VirPool						
18/12/2020	0.00	0.46	0.16	0.13	0.00	0.24
27/12/2020	0.09	0.06	0.10	0.51	0.01	0.23
17/01/2021	0.52	0.28	0.09	0.02	0.00	0.08
03/02/2021	0.84	0.08	0.01	0.02	0.00	0.05
24/02/2021	0.86	0.04	0.00	0.10	0.00	0.00
VaQuERo						
18/12/2020	0.00	0.42	0.12	0.11	0.00	0.35
27/12/2020	0.20	0.15	0.00	0.41	0.00	0.24
17/01/2021	0.51	0.23	0.09	0.05	0.00	0.12
03/02/2021	0.70	0.21	0.00	0.09	0.00	0.00
24/02/2021	0.94	0.00	0.00	0.00	0.00	0.06

Table S5: Frequencies of selected alleles in the Austrian wastewater sample from February 24, 2021. We have selected all alleles which had in our variant profile probability less that 1% for both B.1.1.7 and B.1.160 and probability at least 25% in B.1.258. We also display the number and fraction of reads containing this allele and the frequencies from these profiles. The first seven rows support a high percentage of B.1.258 variant.

			Frequency in			
Pos	Allele	# reads	reads	B.1.1.7	B.1.160	B.1.258
20268	G	245	1.000	0.000	0.001	0.993
829	T	18060	0.315	0.001	0.001	0.273
18028	T	8005	0.304	0.000	0.001	0.789
17104	T	17743	0.288	0.000	0.002	0.996
23876	A	2007	0.272	0.000	0.000	0.298
22127	T	4922	0.230	0.000	0.000	0.300
25947	T	1922	0.171	0.000	0.001	0.298
12988	T	25	0.002	0.000	0.001	0.790
7767	C	2	0.002	0.000	0.002	0.992
14697	T	18	0.001	0.000	0.000	0.281
22879	A	36	0.001	0.000	0.001	0.991
26972	C	55	0.001	0.000	0.002	0.783
27800	A	26	0.001	0.000	0.001	0.849
28083	T	123	0.001	0.000	0.007	0.265
29734	C	86	0.001	0.000	0.002	0.976
15598	A	0	0.000	0.000	0.002	0.790
16394	T	1	0.000	0.002	0.001	0.299
18176	T	4	0.000	0.000	0.000	0.284
19398	T	0	0.000	0.000	0.004	0.297
20451	T	0	0.000	0.000	0.001	0.365
24910	C	56	0.000	0.000	0.002	0.791
8047	T	0	0.000	0.000	0.001	0.993

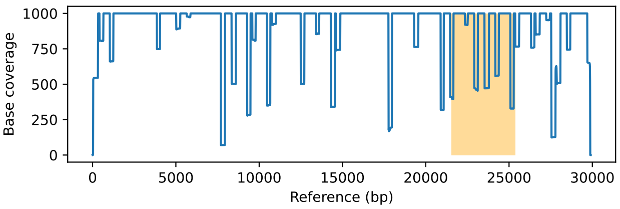

(a) ONT short, clinical sample ERR4868988 (average coverage: 1920, median coverage: 1818)

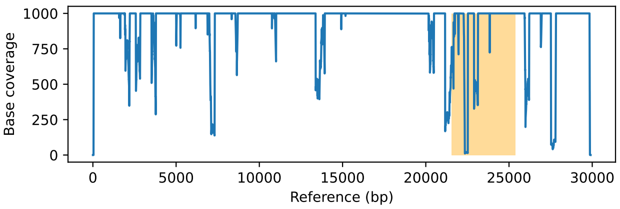

(b) Illumina, clinical sample ERR4839756 (average coverage: 4527, median coverage: 3810)

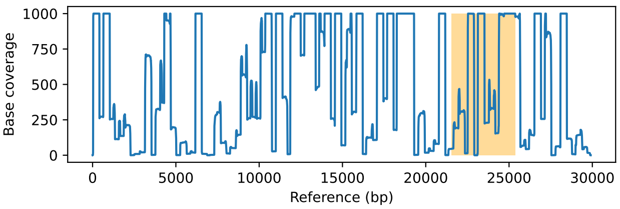

(c) ONT short, wastewater sample SRR15275979, (average coverage: 1057, median coverage: 302)

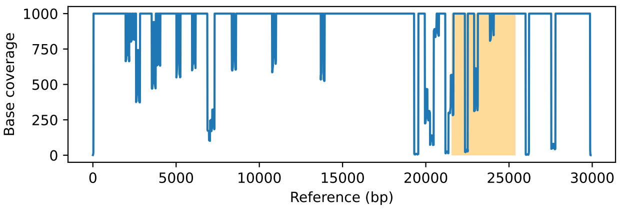

(d) Illumina, wastewater sample CoV-3813-S77797-npa (average coverage: 26223, median coverage: 11579)

Figure S1: Typical coverage along the genome in clinical single-patient and wastewater samples. The y-axis is truncated at coverage of 1000. In wastewater samples, the coverage is much more variable with many regions of extremely low coverage. The Spike gene region, which contains many variant segregating mutations, is highlighted in orange.

Table S6: Frequencies of selected alleles in the Nice wastewater samples from October 2020. We have selected all alleles which had in our variant profile probability less that 1% for both B.1.160 and "other" and probability at least 90% in B.1.1.7. We display the fraction of reads containing each allele in the samples from the five locations where VirPool estimated at least 7% of the Alpha variant. Positions where at least one of the five samples have allele proportion at least 5% are highlighted in boldface.

		Frequency in				
Pos	Allele	Fabron	Madeleine	Harbor	Paillon	East Nice
**913**	T	0.145	0.106	0.121	0.095	0.132
3267	T	0.034	0.035	0.034	0.042	0.042
5388	A	0.000	0.000	0.009	0.000	0.009
**5986**	T	0.062	0.000	0.006	0.014	0.022
6954	C	0.000	0.000	0.000	0.000	0.000
11296	G	0.006	0.003	0.006	0.008	0.002
**14676**	T	0.342	0.379	0.399	0.421	0.358
**15279**	T	0.121	0.163	0.212	0.179	0.114
16176	C	0.015	0.003	0.009	0.008	0.007
**23063**	T	0.000	0.333	0.000	0.000	0.000
23271	A	0.023	0.011	0.018	0.014	0.020
**23709**	T	0.028	0.000	0.078	0.060	0.152
**24506**	G	0.209	0.224	0.019	0.168	0.077
**24914**	C	0.156	0.005	0.001	0.000	0.045
**27972**	T	0.062	0.000	0.037	0.037	0.027
28048	T	0.000	0.000	0.010	0.000	0.000
28111	G	0.017	0.019	0.014	0.012	0.015
**28280**	C	0.169	0.236	0.229	0.209	0.214
**28281**	T	0.158	0.214	0.208	0.185	0.194
**28282**	A	0.162	0.221	0.222	0.195	0.205
**28977**	T	0.000	0.458	0.000	0.000	0.026

Table S7: Frequencies of selected alleles in the Nice wastewater samples from March 2021, Harbor location. The top part of the table contains all alleles which had in our variant profile probability less that 1% for B.1.160, B.1.1.7 and "other" and probability at least 90% in B.1.177. Similarly, the bottom part contains all alleles with probability at least 90% in B.1.160 and less than 1% in B.1.177, B.1.1.7 and "other". We display the count and fraction of reads containing each allele in this sample as well as allele probabilities in these profiles.

			Frequency in				
Pos	Allele	# reads	reads	B.1.177	B.1.160	B.1.1.7	other
22227	T	268	0.691	0.995	0.006	0.001	0.004
28932	T	6	0.085	0.996	0.002	0.000	0.001
6286	T	19	0.022	0.995	0.004	0.001	0.002
29645	T	3	0.008	0.995	0.003	0.000	0.001
445	C	3	0.004	0.994	0.003	0.000	0.001
21255	C	0	0.000	0.989	0.002	0.001	0.002
26801	G	0	0.000	0.979	0.003	0.000	0.001
26876	C	205	0.274	0.001	0.987	0.000	0.000
4543	T	18	0.205	0.004	0.987	0.003	0.003
13993	T	2	0.105	0.001	0.990	0.000	0.000
16889	G	1	0.100	0.000	0.988	0.000	0.000
17019	T	1	0.100	0.001	0.987	0.002	0.001
5629	T	3	0.088	0.001	0.962	0.000	0.001
15766	T	30	0.037	0.001	0.988	0.000	0.004
11497	T	1	0.004	0.001	0.988	0.002	0.000
25710	T	0	0.000	0.001	0.988	0.001	0.002
28975	C	0	0.000	0.001	0.985	0.000	0.000
29399	A	0	0.000	0.001	0.987	0.000	0.000
9526	T	0	0.000	0.001	0.989	0.000	0.001

References

Amman F, Markt R, Endler L, Hupfauf S, Agerer B, Schedl A, Richter L, Zechmeister M, Bicher M, Heiler G, Triska P, Thornton M, Penz T, Senekowitsch M, Laine J, Keszei Z, Klimek P, Nagele F, Mayr M, Daleiden B, Steinlechner M, Niederstatter H, Heidinger P, Rauch W, Scheffknecht C, Vogl G, Weichlinger G, Wagner AO, Slipko K, Masseron A, Radu E, Allerberger F, Popper N, Bock C, Schmid D, Oberacher H, Kreuzinger N, Insam H, Bergthaler A (2022) Viral variant-resolved wastewater surveillance of SARS-CoV-2 at national scale. Nature Biotechnology Accepted. Online ahead of print.
